# The Implications of Cytochrome P450 2D6/CYP2D6 Polymorphism in the Therapeutic Response of Atypical Antipsychotics in Adolescents with Psychosis—A Prospective Study

**DOI:** 10.3390/biomedicines12030494

**Published:** 2024-02-22

**Authors:** Adriana Cojocaru, Adina Braha, Roxana Jeleriu, Nicoleta Ioana Andreescu, Maria Puiu, Luminita Ageu, Roxana Folescu, Carmen Lacramioara Zamfir, Laura Alexandra Nussbaum

**Affiliations:** 1 Department of Neurosciences, “Victor Babes” University of Medicine and Pharmacy, 2 Eftimie Murgu Square, 300041 Timisoara, Romania; adriana.cojocaru@umft.ro (A.C.); nussbaum.laura@umft.ro (L.A.N.); 2 Doctoral School, “Victor Babes” University of Medicine and Pharmacy, 2 Eftimie Murgu Square, 300041 Timisoara, Romania; 3 Department of Second Internal Medicine-Diabetes, Nutrition, Metabolic Diseases, and Systemic Rheumatology, “Victor Babes” University of Medicine and Pharmacy, 300041 Timisoara, Romania; 4 Department of Microscopic Morphology, Discipline of Genetics, Genomic Medicine Centre, “Victor Babeș” University of Medicine and Pharmacy, 2 Eftimie Murgu Square, 300041 Timisoara, Romania; jeleriu.roxana@yahoo.com (R.J.); andreescu.nicoleta@umft.ro (N.I.A.); maria_puiu@umft.ro (M.P.); 5 Mental Health Department for Children and Adolescents, “Louis Turcanu” Children’s Emergency Hospital, 2 Doctor Iosif Nemoianu, 300011 Timisoara, Romania; lumiageu@yahoo.com; 6 Department of Balneology, Medical Recovery and Rheumatology, Family Medicine Discipline, Center for Preventive Medicine, Center for Advanced Research in Cardiovascular Pathology and Hemostaseology, “Victor Babes” University of Medicine and Pharmacy, 2 Eftimie Murgu Square, 300041 Timisoara, Romania; folescu.roxana@umft.ro; 7 Department of Morpho-Functional Sciences I, “Grigore T. Popa” University of Medicine and Pharmacy, 16 Universitatii Street, 700115 Iasi, Romania; carmen.zamfir@umfiasi.ro

**Keywords:** atypical antipsychotics, cytochrome CYP2D6, adolescents, PANSS score, adverse reaction

## Abstract

Background: The plasma level of antipsychotics and their metabolites depends on the activity of the cytochrome P450 (CYP) system in the liver. This research aims to test the individual response variability to atypical antipsychotic drugs, depending on the activity of the CYP2D6 enzyme. Methods: In a prospective, noninterventional study, we included 56 adolescents, 51.79% male, diagnosed with schizophrenia. The patients underwent DNA sampling for genotyping SNP by RT-PCR and CYP* allelic variants using Applied Bio-systems™ TaqMan^®^ Assays Foster City, CA, USA). and clinical and paraclinical assessments. The effectiveness of the therapy was evaluated with the PANSS scores at baseline and 3, 6, and 12 months after the initiation of an atypical antipsychotic treatment. Results: Based on the genotyping results, the patients were divided into slow metabolizers (Group 1), extensive metabolizers (Group 2), and intermediate metabolizers (Group 3). The PANSS score showed a significant decrease in Group 2, compared to Group 3 after 3 (*p* = 0.02), 6 (*p* = 0.0009), and 12 months (*p* < 0.0001). The patients in Group 1 showed high PANSS scores, and those in Group 2 had fewer adverse reactions than the other groups. Conclusions: Assessing the CYP2D6 polymorphism may be useful in clinical pediatric psychiatric practice towards improving clinical results and patients’ quality of life.

## 1. Introduction

Psychotic disorders represent a polymorphic group of psychiatric diseases characterized by an impairment of thought and perception, a loss of contact with reality, and atypical behavior with the emergence of difficulty in performing daily tasks and maintaining proper social interactions. Psychoses lead to a disarray of the entire personality, which makes room for behaviors and attitudes inconsistent with the child’s biological age. As a psychotic disorder, schizophrenia is clinically characterized by positive symptoms (illusions, hallucinations, disordered thoughts) and negative symptoms (affective flattening, alogia, avolition, anhedonia, catatonia, and social isolation). This disorder has a chronic evolution but follows a relapse–remission pattern with disability accumulated over time. When an acute episode happens in remission, patients face negative symptoms and cognitive impairment [1,2,3,4,5,6].

The more commonly used antipsychotics to treat psychotic symptoms in adolescence are typical, first-generation (e.g., Haloperidol, Levomepromazine, Flupentixol) or atypical, second-generation drugs (e.g., Risperidone, Olanzapine, Quetiapine, Aripiprazole) [7,8].

Typical antipsychotics can lead to adverse effects like extrapyramidal effects, malignant neuroleptic syndrome, sedation, epileptogenic effects, cardiovascular dysfunction, peripheric or central anticholinergic dysfunctions, and endocrinologic dysfunctions, as well as hematological, ophthalmological, and dermatological effects [9,10,11,12,13,14,15,16]. There is a smaller risk regarding adverse effects in the case of atypical antipsychotics. However, there is a higher tendency towards weight gain, metabolic syndrome occurrence, hyperinsulinemia, dyslipidemia, and diabetes mellitus [17,18,19]. The studies published to date regarding the adult population seem to support the increased efficacy and tolerability of atypical antipsychotics compared with typical ones.

However, studies regarding children and adolescents are limited. The therapeutic effects of and adverse reactions to atypical antipsychotics arise as a consequence of their action on different types of receptors: dopaminergic, serotoninergic, muscarinic, adrenergic, or histaminic.

The metabolism of antipsychotics takes place in the liver under the action of cytochrome P450 through processes of oxidative dealkylation and the reduction of the ketone group. The plasma level of antipsychotics and their metabolites depends on the activity of the cytochrome P450 system in the liver, which can intensify or lower their metabolism [20,21].

*CYP genes* encode P450 proteins, which are enzymes involved in catabolism (oxidation, reduction). More than 70 polymorphisms, allelic variants associated with increased, decreased, or absent enzyme activity, have been described. The most common allelic variants in the literature are CYP2D6*4, CYP2D6*5, CYP2D6*10, and CYP2D6*17 [22].

Interindividual variations in the plasma level of antipsychotics may arise due to differences in the gene polymorphism contribution to the enzymes that metabolize the antipsychotic drug [23]. CYP2D6 is a cytochrome P450 enzyme involved, to a different extent, in the metabolism of most antipsychotics. Enzyme polymorphism divides antipsychotic metabolizers into slow, intermediate, extensive, and ultrarapid [22].

Slow metabolizers show homozygosity or heterozygosity for inactive alleles, which leads to decreased enzyme activity or an overall lack of enzyme and may cause an increased risk of adverse effects and toxin accumulation. There is also an increased risk of overdose due to inactive or dysfunctional genes. Intermediate metabolizers show diminished enzymatic activity due to some allelic variants that produce mutant proteins. Extensive metabolizers have wild-type allelic variants that exhibit normal enzyme activity and present a risk of underdosing, thus requiring increased doses. Ultra-rapid metabolizers have increased enzyme activity due to the overexpression of the metabolizing protein secondary to duplications or mutations of the *CYP2D6 gene*. In this case, a duplicated CYP450 enzyme gene increases the risk of therapeutic inefficiency [20,22,24,25].

Commonly used antipsychotics are metabolized by the CYP enzyme in the liver [21]. Risperidone is extensively metabolized, mainly through 9-hydroxylation by the CYP2D6 enzyme [26]. Aripiprazole is extensively metabolized by mainly CYP2D6 and CYP3A4, usually with a half-life of about 75 h for Aripiprazole. However, in individuals with poor CYP2D6 activity (poor metabolizers), the mean half-life is about 146 h [27,28].

Olanzapine is predominantly bound to albumin and metabolized mainly by CYP1A2 and, to a lesser extent, via CYP2D6, a minor pathway [29].

This study aimed to investigate the relationship between the CYP2D6 polymorphism and optimal clinical results in adolescents with psychosis. The primary outcome consisted of the patients’ clinical improvement, reflected by the Positive and Negative Syndrome Scale (PANSS), the “gold standard” for the assessment of antipsychotic treatment efficacy [30], and the secondary outcome was adverse effects.

## 2. Materials and Methods

In a prospective, noninterventional, observational study, we enrolled 69 patients diagnosed with schizophrenia according to the DSM5 criteria [31], admitted to the Clinical Department of Pediatric Psychiatry from the Emergency Clinical Hospital for Children “Louis Ţurcanu”, Timișoara, the main provider of pediatric medical services in the western part of Romania, between the years 2017 and 2020. Patients and their parents/legal guardians signed an informed consent form prior to study inclusion. The study followed the Regulation of the Ethics Committee of the “Victor Babeş” University of Medicine and Pharmacy of Timișoara and the Emergency Clinical Hospital for Children “Louis Ţurcanu”, Timișoara, Romania, respectively, the GCP (Good Clinical Practice) requirements (13/8 January 2017).

### 2.1. Inclusion and Exclusion Criteria

A total of 56 patients were included in the final analysis based on the following inclusion criteria:

- The presence of a schizophrenia diagnosis by the DSM 5 [31], using the psychiatric interview and the K-SADS-PL interview (Scale for Affective Disorders and Schizophrenia, version for children aged 6 to 18) as a reference [32];
- An indication for treatment with one of the following atypical antipsychotics—risperidone, aripiprazole, or olanzapine;
- Aged between 13 and 18;
- A PANSS score ≥ 70.

Thirteen patients were excluded from the study: seven did not meet the age criteria, and six had a PANSS score ≤ 70.

### 2.2. Patient Medication

The atypical antipsychotics used were risperidone (2–4 mg/day), aripiprazole (10–15 mg/day), and olanzapine (5–10 mg/day), which, statistically, are found most frequently in the treatment scheme of patients with psychosis. Each patient’s attending physician administered the medication, and there was no intervention in the prescription of the treatment, the attending physician being the one who administered another antipsychotic when the evolution was not favorable or adverse effects appeared, according to the physician’s clinical judgment. In 38% of the patients, two or even three antipsychotic changes were necessary due to the occurrence of adverse effects or the persistence of psychotic symptoms. In some cases, two antipsychotics were administered successively (risperidone with aripiprazole or risperidone with olanzapine). In most cases, risperidone was replaced with olanzapine.

### 2.3. Clinical Assessments

Prior to initiating the medication, the patients underwent clinical and paraclinical assessments: vital signs (pulse, blood pressure, temperature), body weight, height, and the calculation of the body mass index (BMI); and extensive laboratory tests, for example, complete blood count, electrolytes, urea, serum creatinine, liver transaminases, thyroid function, fasting glycemia and insulin, electroencephalogram (EEG), and magnetic resonance imaging (MRI). The vital signs, body weight, BMI, and laboratory values were monitored dynamically throughout the study. The BMI is presented only as a percentage increase compared to the normal values according to gender and age.

Clinical assessments of the patients were carried out by interview and the use of scales such as PANSS, UKU (Adverse Events and Symptoms) [33], C-SSRS (Columbia Suicidality Severity Rating Scale) [34], Barnes Akathisia Rating Scale (BARS) [35], K-SADS-PL (Scale for Affective Disorders and Schizophrenia) [32], Diagnostic and Statistical Manual of Mental Disorders (DSM 5) [31], and GAF Score (Global Assessment of Functioning) [36]. The adverse events and symptoms are presented as a percentage of cases in the study group.

The PANSS assessed 7 positive items, 7 negative items, and 16 items about general psychopathology, with a minimum score of 30 and a maximum of 210 [37].

To analyze the genetic peculiarities regarding the patients’ response to pharmacotherapy, with increased safety, tolerability, and maximum efficiency of therapy with atypical antipsychotics, we performed DNA sampling for genotyping *SNP* by RT-PCR and CYP* allelic variants determined by allelic-specific fluorescence measurement using allelic discrimination software (Quant Studio 6, version 1.6.1., Applied Biosystems™ TaqMan^®^ Assays, Foster City, CA, USA).

### 2.4. Study Outcomes

The effectiveness of the therapy was evaluated by monitoring the changes in the average PANSS scores, evaluated at different time intervals as follows: T0—initial moment, before the administration of the pharmacotherapy; T1—3 months after the treatment onset; T2—6 months after the treatment initiation; and T3—12 months after the initiation of treatment. Also, adverse reactions and symptoms were monitored.

#### Statistical Analysis

The information collected in this study was statistically processed using Med-Calc^®^ Statistical Software version 20.014 and Microsoft Excel. The distribution of continuous variables was tested with the Shapiro–Wilk test. Normally distributed variables are presented as means and standard deviations. For descriptive statistics, the results are presented as absolute frequencies and percentages from the subgroups’ total for nominal or dichotomous variables, respectively. To compare the PANSS score at different time points or between different genotype groups, we used a repeated-measures ANOVA Bonferroni-corrected with and without grouping variables and drew graphical representations. To compare the differences in PANSS scores between 2 groups, we used a t-test for means. A *p*-value < 0.05 was considered significant for the statistical tests.

## 3. Results

After applying the inclusion and exclusion criteria, 56 patients between 13 and 18 years old, with a mean age of 15.1 ± 2 years, were included in the analysis. Most subjects were male, with a proportion of 51.7% (29/56). The average PANSS score recorded for the study group was 119.8 ± 32.4, with a positive symptoms score of 23.8 ± 6.5 and a negative one of 20 ± 8.8.

The patients were divided into three groups based on the pharmacogenetic tests:

- Group 1 (*SNP genotype*) included 3.6% (2/56) of the patients: slow metabolizers who have the AL-2 allele (CYP2D6*4) and do not have the functional AL-1 allele;
- Group 2 (CYP2D6*WT) included 69.6% (39/56) of the patients: extensive metabolizers carrying the functional AL-1 allele;
- Group 3 (WT/*4 genotype) included 26.8% (15/56) of the patients: intermediate metabolizers carrying a functional allele (WT) and a nonfunctional allele (*4).

The general characteristics, genotypes, and therapy at baseline and after the pharmacogenetic test of the studied patients are presented in Table 1.

### 3.1. Clinical Efficacy of Atypical Antipsychotics Assessed Using the PANSS Score Based on the CYP2D6 Genotype

After the initiation of the treatment, the clinical status improved, which was reflected throughout the following clinical interviews. There was a decrease in the global PANSS score among the entire group of patients included in the study, with a mean score difference of 37.1 at 12-month follow-up (*p* < 0.0001, Table 2).

**Figure 1:**
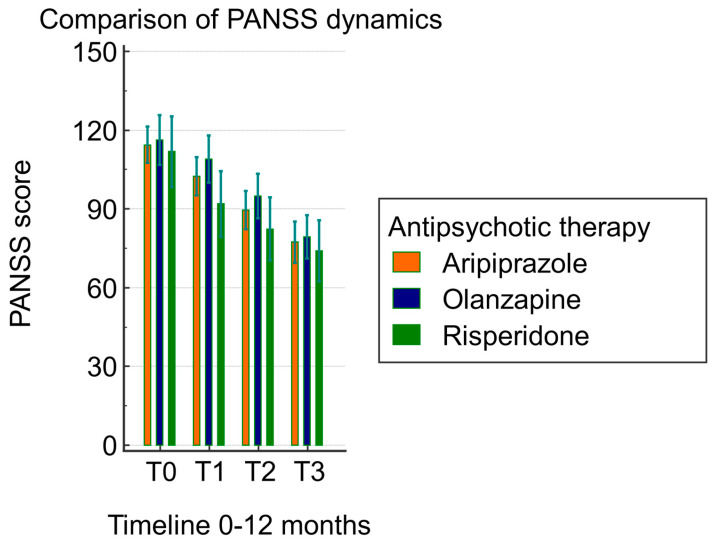
Comparison of PANSS score dynamics according to antipsychotic therapy.

The repeated-measures ANOVA performed with antipsychotic therapy as a grouping variable indicated an epsilon of 0.567 (Greenhouse–Geisser). There were no significant differences in the PANSS score dynamics between the groups treated with aripiprazole, risperidone, and olanzapine (*p* = 0.3). In Table 3, the source of variation attributed to the antipsychotic therapy within-subject, group, and factor interactions is displayed. According to the Greenhouse correction factor applied to the 1.7 degrees of freedom, there were significant differences in the PANSS score between the measurements at different timepoints (*p* < 0.001). However, the differences observed were not related to the group membership (*p* > 0.05) (Table 3, Figure 1).

**Figure 2:**
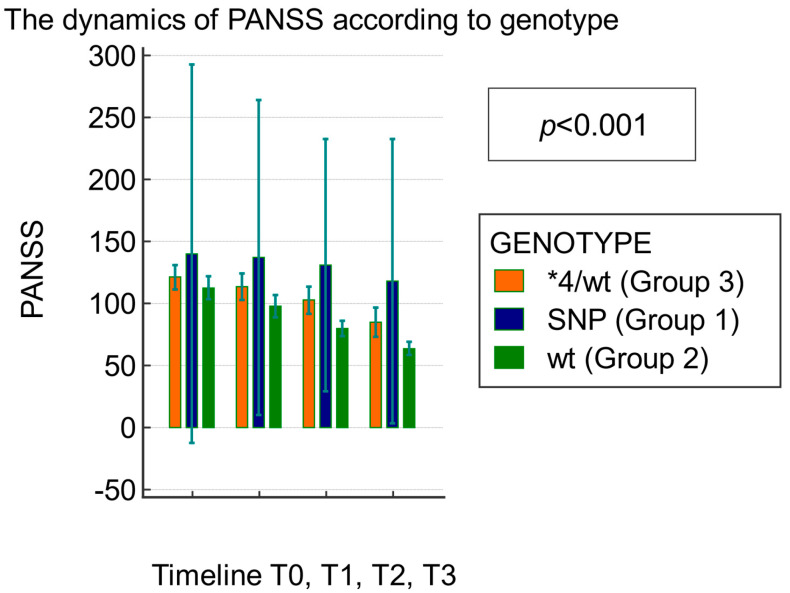
The dynamics of PANSS score during the 12-month follow-up according to genotype.

Similarly, the repeated-measures ANOVA performed with the SNP genotype as a grouping factor indicated a Greenhouse–Geisser epsilon of 0.763. There were significant differences in the PANSS score dynamics between the groups with CYP2D6*4 (Group 1), CYP2D6*WT (Group 2), and WT/*4 (Group 3). Table 4 displays the source of variation attributed to the SNP genotype within -subject, group, and factor interactions. According to the Huynh–Feldt correction factor applied to the 2.4 degrees of freedom, there were significant differences in the PANSS score between measurements at different timepoints (*p* < 0.001) and group membership (*p* = 0.001) (Table 4, Figure 2).

The mean PANSS scores at baseline were similar in Group 2 and Group 3 (*p* = 0.30, Table 5). However, the PANSS score showed a significant decrease in patients with the *WT* genotype (Group 2) compared to the WT/*4 genotype (Group 3) at T1 (*p* = 0.02), T2 (*p* = 0.0009), and T3 (*p* < 0.0001), indicating clinical improvement, with a favorable response to medication (Table 4). In Group 2, the initial PANSS score decreased significantly at the 12-month follow-up by 37.10% (116.70 ± 25.16 versus 75.60 ± 12.40, *p* < 0.0001) after the atypical antipsychotic treatment. The results are interpreted as clinically significant when the decrease in the PANSS score is more than 30% [38]. These findings indicate that the patients with the mixed WT/*4 genotype (Group 3) responded less effectively to medication, with higher PANSS scores correlated with unfavorable clinical evolution. The patients with the *SNP* genotype (Group 1) did not show significant clinical improvement and showed high PANSS scores (*p* > 0.05, Figure 2). The resulting implications of the *CYP2D6* genotype in the evolution of the PANSS score are shown in Table 5 and Figure 2 below.

No statistically significant differences were obtained in the slow and intermediate metabolizer (Table 3) groups when comparing the PANSS scores between the four evaluations. The PANSS score did not significantly decrease after the initiation of the atypical antipsychotic treatment, indicating the low effectiveness of the treatment.

### 3.2. Adverse Reactions Recorded after Atypical Antipsychotic Medication

The effectiveness of the medication in the group of patients with schizophrenia who were administered aripiprazole was greater compared to those who followed treatment with risperidone. In comparison, risperidone was correlated with several adverse effects (weight gain, restlessness, anxiety, extrapyramidal symptoms, hyperglycemia, hyperinsulinemia, and orthostatic hypotension). Overall, 26.8% (15/56) of the patients who underwent treatment with risperidone required a change in medication due to the adverse effects. Overall, some of the patients had multiple adverse effects. The most frequently encountered were weight gain, hyperprolactinemia, galactorrhea, extrapyramidal symptoms, and headaches.

A percentage of 37.5% (21/56) of the patients included in the study had adverse reactions. The incidence of adverse effects is presented in Table 6.

Increased adverse reactions from baseline (T0) were seen in patients in Group 1 and Group 3 (*p* < 0.001, α = 0.001). The least common adverse effects in the evaluated group of patients were insomnia, hyper/hypothyroidism, headaches, drowsiness, weight loss, and, in terms of biological changes, the occurrence of anemia and increased bilirubin or uric acid. The most frequent biological changes were increased body mass index, hyperglycemia, hyperprolactinemia, dysmenorrhea, and gynecomastia.

## 4. Discussions

The present study evaluated the cytochrome P450 2D6/CYP2D6 polymorphism in a pediatric Romanian population previously diagnosed with schizophrenia and treated with antipsychotics (aripiprazole, risperidone, and olanzapine) in different combinations based on the specialty guidelines and clinical judgment of their physician. We identified three different genotypes: the *SNP genotype* with the AL-2 allele (CYP2D6*4) and without the functional AL-1 allele, which indicates a slow metabolizer; the CYP2D6*WT genotype, found in the majority of our pediatric patients with schizophrenia, has extensive metabolic activity, carrying the functional AL-1 allele; and last, we identified the WT/4* genotype in about 27% of the patients, which has intermediate metabolism, carrying a functional allele (*WT*) and a nonfunctional allele (*4). Our findings indicated that patients with CYP2D6*WT (extensive metabolizers) had the best therapeutic response, reflected by a significantly reduced PANSS score at the 12-month follow-up of 75.60 ± 12.40, compared to patients with WT/4*, who presented a less favorable PANSS score of 106.9 ± 26.84. An improvement of ≥30% in the PANSS score from baseline is considered a good response to treatment [38]. The decrease in the PANSS scores in our study group exceeds the threshold of 30%. The majority of the patients with CYP2D6*WT were treated with aripiprazole. The patients without the functional AL-1 allele did not respond favorably to medication. An explanation for these findings is that extensive metabolizers are carriers of two functional alleles, and intermediates have only one functional allele. It is known that intermediate metabolizers have reduced enzyme activity, and extensive metabolizers have normal enzyme activity.

Pharmacogenetic tests targeting polymorphisms in the CYP family represent investigations that may indicate the choice of the most optimal treatment and its appropriate dosage based o a patient’s age, associated comorbidities, and intensity of symptoms [25]. *CYP2D6* genotyping classifies patients into extensive, poor, intermediate, and ultrarapid metabolizers according to their phenotype. The *CYP2D6* gene is polymorphic with numerous alleles; alleles with reduced enzyme activity will result in slow (homozygotes) or intermediate (heterozygotes) metabolic phenotypes; an ultrafast metabolic phenotype is found in the case of duplications of the *CYP2D6* gene [39]. In patients with *SNP* or mixed *WT/SNP* polymorphisms accompanied by poor metabolizers, it is recommended to avoid the administration of Haloperidol, risperidone, and aripiprazole, with these being replaced by olanzapine, Quetiapine, or clozapine [40].

Due to the FDA approval of aripiprazole for *CYP2D6* poor metabolizers and the lack of recommendations for intermediate metabolizers, Xiaodan Zhang reviewed studies looking at the association between aripiprazole and CYP2D6 polymorphisms published up to February 2018 and concluded that the serum concentration of aripiprazole was significantly different between extensive and intermediate metabolizers but not between poor metabolizers; therefore, further randomized studies are needed to demonstrate the clinical significance of this [41].

In a meta-analysis of eight randomized clinical trials involving 457 pediatric participants, no significant differences were found in the mean PANSS score or positive or negative symptom scores between the olanzapine and risperidone treatment groups. However, whether the clinical efficacy of olanzapine for the treatment of children and adolescents with psychosis is equivalent to or superior to risperidone will need to be confirmed with a larger sample [42]. However, the contribution of *CYP2D6* in the biotransformation of olanzapine is minimal, as this antipsychotic is mainly metabolized via CYP1A2 and UGT1A4 [43].

Antipsychotic treatment is now associated with a higher risk of diabetes onset, which raises even greater interest in monitoring and research using various methods to decrease this risk rate [44]. This subject is broader because patients with schizophrenia are, in any case, associated with an increased risk of developing diabetes compared to the general population. This risk increases with the implementation of antipsychotic treatment. Most first-generation antipsychotics, as well as olanzapine and clozapine, have been shown to impair glycemic control [10,11,17,44,45,46,47]. Other second-generation (or atypical) antipsychotics such as amisulpride, ziprasidone, and aripiprazole appear less associated with this risk. However, they are still known to alter glucose metabolism and increase the risk of metabolic syndrome [10,44,46,47,48].

In our group, 14.28% of the patients presented with increased blood glucose. However, no patients developed diabetes. Moreover, the follow-up duration was short. Therefore, further studies with a longer follow-up duration are needed to confirm this hypothesis.

The polymorphic variation in *CYP2D6* has been associated with increased prolactin in the case of treatment with atypical antipsychotics [49]. In the evaluated group, a prevalent adverse effect of the medication was hyperprolactinemia. A percentage of 25% of patients who suffered from adverse effects had elevated prolactin levels. A previous study found an association between prolactin concentrations and *CYP2D6* polymorphisms in 47 pediatric patients with autism spectrum disorders or conduct disorders receiving risperidone, aged 10–19, showing higher prolactin levels in patients with reduced or no activity of CYP2D6 versus normal metabolizers [50]. A possible hypothesis may explain the interactions of the 5-methoxy tryptamine (5MT), *CYP2D6*, serotonin, and dopamine systems with prolactin release from the pituitary [51]. With the properties of *CYP2D6* poor metabolizers, impaired metabolic function may result in reduced serotonin reproduction by 5MT. As a result, this may lead to a higher dopamine tone in the anterior pituitary, as serotonin generally exerts an inhibitory effect on dopamine pathways. Consequently, a prolactin response could be observed in *CYP2D6* poor metabolizers after dopamine antagonist treatment, such as risperidone [52].

In addition, a study by Bushe and Shaw in 2007 on two groups of patients showed that the incidence of hyperprolactinemia is increased in patients with schizophrenia, especially hospitalized patients [53]. There are no known data on the impact of CYP2D6 enzyme polymorphisms on short- and long-term adverse effects in children and adolescents with psychoses who are being treated with atypical antipsychotic drugs. However, the pharmacokinetics of atypical antipsychotics are influenced by age and the period of neurodevelopment, but the half-life also varies depending on the activity of the CYP2D6 enzyme [49,54]. However, the effects of CYP2D6 genetic polymorphisms on the serum prolactin concentration are still controversial. This may relate to methodological differences (e.g., open-label retrospective, prospective, and case–control studies) or small sample sizes [53].

Weight gain results in reduced patient compliance, regardless of symptomatic improvement. Weight gain is one of the most important side effects of risperidone. Regarding the *CYP2D6* genotype, Nussbaum et al. noted that patients with the *1/*4 genotype (intermediate metabolizer phenotype) had significantly higher weight gain values than patients who did not carry the *4 allele in a study on children and adolescents being treated with antipsychotics (risperidone, aripiprazole, or olanzapine) [25]. Vicki et al. reported that patients treated with atypical antipsychotics who had the *CYP2D6* genotype *1/*3 or *4 had a significantly greater percent change in body mass index than those with the *1/*1.76 genotype (*p* < 0.009) [55]. Lane et al. found a significant association between the CYP2D6*10 allele and weight gain in patients aged 18–60 undergoing risperidone treatment. In this study, patients with the WT/*4 genotype, intermediate metabolizers, had significantly higher weight gain values than patients without the nonfunctional *4 allele, suggesting that high concentrations of risperidone result in increased exposure, which may trigger risperidone-induced weight gain and metabolic effects [56]. The *CYP2D6* genotype in children and adolescents could be a good predictor of responses to risperidone, and side effects could be recorded. Therefore, pharmacogenetic screening would be useful for future clinical practice, allowing personalized treatment, especially for at-risk people, such as those with metabolic risk.

Among the cohort studies identified, Jallaq et al. (2021) reported that *CYP2D6* phenotypes are significantly associated with a change in the BMI percentile (*p* = 0.03) in children and adolescents ± 18 years old taking aripiprazole, also depending on the duration of the treatment and the number of CYP2D6 substrate-associated medications [57]. In another study (Correia et al., 2010) in children and adolescents aged 8.6 ± 4.3 taking risperidone, there was a significantly smaller increase in BMI of 4.8% and waist circumference of 5.8% in ultrarapid metabolizers compared to normal metabolizers, supporting a link between *CYP2D6* metabolic phenotypes and antipsychotic-induced weight. However, this study’s results are contradictory, possibly due to the small number of poor metabolizer subjects whose waist circumference increase appeared smaller than that of normal metabolizers by 4% [58]. Another study that included 50 children and adolescents aged 16 ± 1.25 with schizophrenia at the time of their first psychotic episode showed that olanzapine caused more weight gain than quetiapine [59].

Atypical antipsychotics have fewer extrapyramidal neurological adverse effects due to their affinity for dopamine receptors and other receptors that can cause the appearance of other types of adverse reactions (e.g., in addition to an affinity for dopaminergic receptors, they also have serotonergic receptors). Atypical antipsychotics have antagonistic activity for serotonergic, muscarinic, and histamine receptors that can lead to increased body weight, hyperglycemia, and hyperlipidemia [60].

References to the pharmacogenomic biomarker CYP2D6 have been introduced in leaflets for atypical antipsychotics in different sections: clinical pharmacology, mode of administration, precautions, drug interactions, and dosage [61]. Thus, the recommendations for adjusting the dose of atypical antipsychotics, depending on the CYP2D6 phenotype for the antipsychotics taken in the study, are as follows [61]:

- Risperidone, CYP2D6 phenotype—poor, intermediate, and ultrarapid metabolizers—data are insufficient to calculate dose adjustment, but it is recommended to administer other antipsychotics (e.g., olanzapine, quetiapine, clozapine) or to pay close attention to adverse effects and adjust the dose depending on the patient’s clinical response;
- Olanzapine, CYP2D6 phenotype—poor, intermediate, and ultrarapid metabolizers—there are no recommendations for adjusting the therapeutic dose;
- Aripiprazole, CYP2D6 phenotype—intermediate and ultrarapid metabolizers. There are no recommendations for adjusting the therapeutic dose. Instead, for poor metabolizers, it is advised to reduce the maximum dose to 10mg/day (67% of the maximum recommended daily dose).

On the other hand, a study on 136 patients, aged 37 ± 13 years, diagnosed with schizophrenia, schizoaffective disorders, delusional disorder, or brief psychotic disorder and treated with a single regimen of risperidone showed no association between CYP2D6 polymorphisms (*1/*1, *1/*10, and *10/*10) and clinical improvement outcomes based on PANSS. Furthermore, there was no correlation between the plasma concentrations of the active moiety (risperidone and 9-hydroxy risperidone) and the percent improvement in PANSS, positive, or negative total scores. The study findings suggest that the active fragment’s plasma concentration could only play a role in extrapyramidal adverse reactions [62].

More studies are needed to establish the standardization of genetic testing for antipsychotic drugs and the development of pharmacogenetic protocols depending on the administered antipsychotic drug, taking into account patients’ genetic characteristics.

Depending on the interindividual response, the daily dose of atypical antipsychotics should be adjusted according to the clinical response of patients. Therapeutic drug monitoring (TDM) is an important tool for therapeutic optimization, which can explain either adverse effects or responsiveness in patients treated with a drug with a narrow therapeutic index or multiple drugs. Thus, TDM is crucial in pharmacogenetic testing to optimize a patient’s dose [63].

In our study, CYP2D6 genotyping was important for predicting the clinical evolution of patients undergoing antipsychotic treatment. The results showed statistically significant differences in the clinical scores depending on the type of genotype. The patients presented improvements in symptoms and increased quality of life, demonstrating that a therapeutic intervention based on pharmacogenetic testing leads to an increase in the effectiveness and tolerability of the treatment.

However, the short duration of the study and the small number of patients when comparing the groups by type of genotype are study limitations. However, although the number of participants is relatively small, the Emergency Clinical Hospital for Children “Louis Ţurcanu”, Timișoara, is the main provider of pediatric medical services in the western part of Romania, and between 2017 and to date, there have been 80 pediatric cases of schizophrenia admitted to the hospital; therefore, the number of studied patients is more than 50% of diagnosed cases of schizophrenia. The lack of plasma concentrations of the antipsychotics, at least at the time of final evaluation, is another study limitation. Further, longer-duration studies on large cohorts and randomized clinical trials are needed to validate our research results.

## 5. Conclusions

The high variability in patients’ responses to standard antipsychotic treatment doses, also explained by the differences in the CYP enzyme system, especially the *CYD2D6 gene*, responsible for metabolizing most antipsychotics, justifies the need to administer personalized treatments considering this enzyme system. Determining pharmacological markers for psychosis offers a greater degree of specificity to diagnosis and therapeutic strategies, with much better results in the evolution of the disorder and the reduction of adverse effects. Significant results were obtained in the current study, which may guide the future therapeutic management of psychotic disorders in adolescents.

## Figures and Tables

**Table 1:** General characteristics, genotypes, and therapy of studied patients.

Variable	Value	
Male % (n)	51.7% (29/56)	
Age (years)	15.14 ± 2	
Weight (kg)	69.1 ± 15.6	
Fasting glycemia (mmol/L)	4.5 ± 0.5	
Insulin (µU/mL)	14.2 ± 8.1	
TGO (u/L)	16.3 ± 4.4	
TGP (u/L)	13.1 ± 4.8	
PANSS	119.8 ± 32.4	
Genotype: CYP2D6*4 (Group 1) CYP2D6*WT (Group 2) WT/*4 (Group 3)	3.6% (2/56) 69.6% (39/56) 26.8% (15/56)	
Antipsychotic therapy	At baseline	After genotyping
Aripiprazole Risperidone Olanzapine	48.2% (27/56) 0% had CYP2D6*4 39.3% had CYP2D6*WT 8.9% had WT/*4 42.8% (24/56) 1.8% had CYP2D6*4 26.8% had CYP2D6*WT 14.2% had WT/*4 9% (5/56) 1.8% had CYP2D6*4 3.6% had CYP2D6*WT 3.6% had WT/*4	58.9% (33/56) 0% 48.2% 10.7% 17.9% (10/56) 1.8% 3.6% 12.5% 23.2% (13/56) 1.8% 17.8% 3.6%

TGO = aspartate aminotransferase; TGP = alanine aminotransferase; PANSS = Positive and Negative Syndrome Scale; CYP2 = cytochrome P450.

**Table 2:** Comparison of PANSS score at the 4 time points for the entire study group.

Factors			Mean Difference	Std. Error	*p* ^a^	95% CI ^a^
PANSS T0	-	PANSS T1	11.815	1.235	<0.0001	8.454 to 15.177
	-	PANSS T2	24.492	1.509	<0.0001	20.383 to 28.602
	-	PANSS T3	37.108	1.920	<0.0001	31.879 to 42.337
PANSS T1	-	PANSS T0	−11.815	1.235	<0.0001	−15.177 to −8.454
	-	PANSS T2	12.677	0.972	<0.0001	10.030 to 15.324
	-	PANSS T3	25.292	1.571	<0.0001	21.014 to 29.571
PANSS T2	-	PANSS T0	−24.492	1.509	<0.0001	−28.602 to −20.383
	-	PANSS T1	−12.677	0.972	<0.0001	−15.324 to −10.030
	-	PANSS T3	12.615	0.967	<0.0001	9.981 to 15.249
PANSS T3	-	PANSS T0	−37.108	1.920	<0.0001	−42.337 to −31.879
	-	PANSS T1	−25.292	1.571	<0.0001	−29.571 to −21.014
	-	PANSS T2	−12.615	0.967	<0.0001	−15.249 to −9.981

^a^ Bonferroni-corrected; T0—initial moment, before the administration of the pharmacotherapy; T1—3 months after the treatment onset; T2—6 months after the treatment initiation; T3—12 months after the initiation of the treatment; N = 56; *p* < 0.05 statistically significant.

**Table 3:** Comparison of PANSS score dynamics according to antipsychotic therapy.

Test of Between-Subject Effects						
Source of Variation		Sum of Squares	DF	Mean Square	F	*p*
Groups (therapy)		2792.316	2	1396.158	1.00	0.374
Test of within-subject effects						
Factor	sphericity assumed	40,892.965	3	13,630.988	220.08	<0.001
	Greenhouse–Geisser	40,892.965	1.7	24,045.001	220.08	<0.001
Group × factor interaction	sphericity assumed	794.166	6	132.361	2.14	0.051
	Greenhouse–Geisser	794.166	3.4	233.484	2.14	0.092

**Table 4:** Comparison of PANSS score in dynamics according to SNP genotype.

Test of Between-Subject Effects						
Source of Variation		Sum of Squares	DF	Mean Square	F	*p*
Groups (genotype)		18,576.847	2	9288.424	10.41	<0.001
Test of within-subject effects						
Factor	sphericity assumed	10,157.967	3	3385.989	63.62	<0.001
	Huynh–Feldt	10,157.967	2.4	4083.290	63.62	<0.001
Group × factor interaction	sphericity assumed	1470.945	6	245.157	4.61	<0.001
	Huynh–Feldt	1470.945	4.9	295.645	4.61	0.001

**Table 5:** Comparison of PANSS score at different time points according to CYP polymorphism in intermediate (WT/*4) and extensive metabolizers (*WT*).

Timeline	Genotype	N	Mean PANSS ± Standard Deviation	*p*
T0	CYP2D6*WT (Group 2)	39	116.70 ± 25.16	0.30
	WT/*4 (Group 3)	15	125.30 ± 33.20	
T1	CYP2D6*WT (Group 2)	39	103.40 ± 23.60	0.02
	WT/*4 (Group 3)	15	122.20 ± 32.62	
T2	CYP2D6*WT (Group 2)	39	90.80 ± 19.40	0.0009
	WT/*4 (Group 3)	15	115.40 ± 30.37	
T3	CYP2D6*WT (Group 2)	39	75.60 ± 12.40	<0.0001
	WT/*4 (Group 3)	15	106.9 ± 26.84	

T0—initial moment, before the administration of the pharmacotherapy T1—3 months after the treatment onset; T2—6 months after the treatment initiation; T3—12 months after the initiation of the treatment; N = 56; *p* < 0.05, statistical significance; *t*-test for means.

**Table 6:** The incidence of adverse effects in the studied group.

Systems/Apparatus Disorders	Side Effects Frequency	Antipsychotic Therapies after Genotyping		
	Overall % (n = 56)	Aripiprazole	Olanzapine	Risperidone
**Gastrointestinal disorders**				
Nausea, vomiting, abdominal pain	10.71% (6/56)	1.78% (1/56)	3.57% (2/56)	5.35% (3/56)
Constipation, diarrhea	19.64% (11/56)	0%	3.57% (2/56)	16.07% (9/56)
Salivary hypersecretion	1.78% (1/56)	0%	1.78% (1/56)	0%
**Nervous system disorders**				
Headache	17.85% (10/56)	0%	5.35% (3/56)	12.5% (7/56)
Extrapyramidal symptoms	17.85% (10/56)	1.78% (1/56)	0%	16.07% (9/56)
Akathisia, tardive dyskinesia	3.57% (2/56)	0%	0%	3.57% (2/56)
Sleepiness	16.07% (9/56)	0%	8.92% (5/56)	7.14% (4/56)
Vertigo	17.85% (10/56)	0%	7.14% (4/56)	10.71% (6/56)
Adverse cognitive effects	10.71% (6/56)	0%	3.57% (2/56)	7.14% (4/56)
**Psychiatric disorders**				
Anxiety	12.50% (7/56)	1.78% (1/56)	1.78% (1/56)	8.92% (5/56)
Agitation	14.28% (8/56)	0%	5.35% (3/56)	8.92% (5/56)
Insomnia	3.57% (2/56)	1.78% (1/56)	1.78% (1/56)	0%
Aggressivity	10.71% (6/56)	0%	5.35% (3/56)	5.35% (3/56)
Suicidal ideation, suicidal attempt	8.92% (5/56)	0%	5.35% (3/56)	3.57% (2/56)
**Metabolic disorders**				
Hyperglycemia	14.28% (8/56)	0%	1.78% (1/56)	12.5% (7/56)
Hyponatremia	1.78% (1/56)	0%	1.78% (1/56)	0%
Increase in BMI (body mass index)	32.1% (18/56)	0%	8.92%(5/56)	23.21% (13/56)
Weight loss ≥ 7%	3.57% (2/56)	1.78% (1/56)	1.78% (1/56)	0%
Weight gain ≥ 7%	10.71% (6/56)	0%	1.78% (1/56)	8.92% (5/56)
**Cardiovascular disorders**				
Tachycardia/Bradycardia	23.21% (13/56)	0%	5.35% (3/56)	17.85% (10/56)
Arrhythmia/QT interval prolongation	1.78% (1/56)	0%	1.78% (1/56)	0%
Hypotension/Hypertension	16.07% (9/56)	0%	5.35% (3/56)	10.71% (6/56)
**Endocrinological disorders**				
Hyperprolactinemia	25.00% (14/56)	0%	7.14% (4/56)	17.85% (10/56)
Hyperthyroidism/Hypothyroidism	3.57% (2/56)	0%	1.78% (1/56)	1.78% (1/56)
**Hematological disorders**				
Anemia	3.57% (2/56)	1.78% (1/56)	1.78% (1/56)	0%
Leukopenia	7.14% (4/56)	0%	1.78% (1/56)	5.35% (3/56)
Neutropenia	7.14% (4/56)	0%	1.78% (1/56)	5.35% (3/56)
Thrombocytopenia	10.71% (6/56)	0%	1.78% (1/56)	5.35% (3/56)
**Gynecological disorders**				
Dysmenorrhea/Amenorrhea	21.42% (12/56)	0%	7.14% (4/56)	14.28% (8/56)
Galactorrhea/Gynecomastia	14.28% (8/56)	0%	5.35% (3/56)	12.5% (7/56)
**Paraclinical analyses**				
Glycosylated hemoglobin increase	10.71% (6/56)	0%	1.78% (1/56)	8.92% (5/56)
Increased creatine phosphokinase	5.35% (3/56)	0%	0%	5.35% (3/56)
Increased triglycerides	8.92% (5/56)	0%	0%	5.35% (3/56)
Increased bilirubin	3.57% (2/56)	0%	1.78% (1/56)	1.78% (1/56)
Increased uric acid	3.57% (2/56)	0%	0%	3.57% (2/56)

## Data Availability

The subjects in this study did not give written consent for their data to be shared publicly, so due to the sensitive nature of the research, supporting data are not available.
